# Striosome‐Like Structural Connectivity Is Reduced in the Rostral Putamen in Anxiety Disorder

**DOI:** 10.1155/da/7682055

**Published:** 2026-04-08

**Authors:** Adrian T. Funk, Jeff L. Waugh

**Affiliations:** ^1^ Division of Pediatric Neurology, University of Texas Southwestern, Dallas, Texas, USA, utsouthwestern.edu; ^2^ Department of Pediatrics and Department of Neurology, University of Texas Southwestern, Dallas, Texas, USA, utsouthwestern.edu

**Keywords:** anxiety, compartment, matrix, striatum, striosome

## Abstract

Anxiety disorders affect roughly a third of adults, and treatment of anxiety‐related symptoms costs more than any other mental health condition. Symptoms often begin in adolescence and are usually lifelong. Identifying shared neuroanatomical features of anxiety is essential for understanding its causes and improving treatments. Developmental abnormalities in the striatum, an obligate node in cortico–striato–thalamo–cortical networks, could predispose to the diverse symptoms of anxiety. The striatum is comprised of two interdigitated tissue compartments, striosome and matrix, that have different embryologic origins, divergent afferent/efferent connectivity, opposing responses to dopamine, and are embedded in distinct structural and functional networks. We used quantitative diffusion tractography to perform connectivity‐based parcellation, identifying striosome‐like and matrix‐like striatal voxels in a 295‐subject cohort (youth‐onset anxiety vs. matched controls). In anxiety, striosome‐like connectivity was depleted and dispersed, especially in a bilateral zone in the rostral putamen. Striosome‐like connectivity was inversely correlated with standardized assessments of anxiety. Matrix‐favoring connectivity was increased in a broad swath of cortico‐striate projections. Increases in the striatal matrix:striosome ratio may predispose individuals to anxiety.

## 1. Introduction

Prior neuroanatomic studies regarding anxiety primarily focused on brain areas associated with limbic functions, including the amygdala, bed nucleus of the stria terminalis, hippocampus, and prefrontal cortex [1, 2]. The striatum, comprised of the caudate and putamen, is the primary input nucleus of the basal ganglia and plays an essential role in decision‐making, movement control, reward processing, and motivation but has been relatively underinvestigated in the predisposition to and progression of anxiety [3]. The composition and connectivity of the striatum are abnormal in individuals with symptoms of anxiety [3, 4]. Stress, a common precipitant of anxiety, leads to a coincident increase in functional activation in the ventral striatum (nucleus accumbens) and potentiates aversive prediction errors [5]. Enhanced functional activation in the striatum was associated with negative behavioral inhibition, a temperament that predisposes to anxiety disorders [6]. Stimulation of projections from the basolateral amygdala to the dorsomedial striatum, a connection that mediates stress‐induced behavior, results in increased anxiety and compulsive grooming (an anxiety‐associated behavior) in mice [7]. Additionally, human anxiety is associated with tissue‐level changes: diminished dopamine receptors in the dorsal and ventral striatum [8].

Striatal projection neurons (SPNs) can be divided into two histologically and neuroanatomically distinct compartments, the striosome and matrix. The striosome is a three‐dimensional, web‐like, labyrinthine structure that spreads throughout the surrounding matrix tissue. The two compartments have distinct properties: they migrate from the lateral ganglionic eminence on different embryologic days [9], have segregated afferent and efferent projections [10–12], are embedded in distinct resting state functional networks [13], and differentially express >60 histochemical markers [14]. The compartments are also selectively susceptible to neurodegenerative and neuropsychiatric disorders, including Huntington disease [15, 16], levodopa‐induced dyskinesia [17], Parkinson disease, drug addiction, and schizophrenia [14].

The striosome and matrix operate in distinct cortico–striato–thalamo–cortical loops [12, 18] and have differential connectivity to cortical and subcortical areas associated with anxiety [19, 20]. There is evidence of compartment‐specific structural connectivity and activation in human and nonhuman subjects with anxiety disorders. Generalized anxiety disorder (GAD) and social anxiety disorder exhibit protein–protein interaction networks unique to either striosome or matrix compartments, respectively [21]. Numerous studies found that striosome‐related circuits support functions that can mitigate or facilitate anxiety, such as motivational control, behavioral flexibility, high‐stress decision‐making, and high risk‐reward cost–benefit motivational conflict [16, 22–24]. For example, in approach‐avoidance conflict conditions (a model for anxiety in both animals and humans) [25], striosome‐selective optogenetic and electrophysiological inhibition blocks cost–benefit decision‐making [23]. Likewise, striosome ablation increases motor vigor, a physical correlate of elevated anxiety in mice [26].

We recently established a method for parcellating the striatum into striosome‐like and matrix‐like compartments in living humans by identifying voxels that exhibit highly biased structural connectivity (as measured by probabilistic tractography) toward striosome‐favoring or matrix‐favoring “bait” regions [27], which were derived from four decades of injected tract tracer studies in animals. Striatal parcellation is highly reliable, with a test–retest error rate of 0.14% [27], and reproduces the relative abundance and spatial distribution of striosome and matrix demonstrated in human tissue [28]. This method replicates the compartment‐specific biases in structural connectivity demonstrated through decades of injected tract tracer studies in animals [29] and is congruent with neuroimaging assessments of human striato–pallido–thalamic [16, 27] and insulo‐striate structural and functional connectivity [18].

We hypothesized that reductions in striosome volume or the strength of cortico‐striosomal structural connectivity could predispose individuals to anxiety disorders. We used connectivity‐based parcellation (probabilistic diffusion tractography) to identify voxels with striosome‐like and matrix‐like patterns of connectivity in a large cohort of young people with moderate or severe anxiety symptoms, compared to matched healthy control subjects. We found that in anxiety subjects, striosome‐like structural connectivity was reduced and shifted in location in a broad striatal distribution, especially in a bilateral zone in the rostral putamen.

## 2. Methods

### 2.1. Participants

All research was conducted according to the principles in the Declaration of Helsinki. This was a secondary analysis of clinical and imaging data collected from multiple institutions and distributed through the National Institute of Mental Health (NIMH) National Data Archive (NDA) [30]. All data collection was approved by Institutional Review Boards for the archiving studies. We included 295 subjects from three prior studies [31–33], matching anxiety and healthy control subjects directly within each study. We matched all control:anxiety pairings for sex and age (within 1 year). We matched for self‐identified race whenever possible (88.16% of matches). For studies A and B, there were an abundance of healthy controls, allowing us to make 2:1 matches (control:anxiety). For study C we matched anxiety and control subjects 1:1. Our combined cohort included 115 anxiety subjects (diagnostic criteria for anxiety described below) and 180 healthy controls (Table 1).

**Table 1:** Age, sex, and self‐identified race for each of the three studies used to generate our anxiety and matched healthy control cohorts.

Study of origin	Age	Sex	Self‐identified race	Anxiety symptom category
Study A	Controls: 18–19 years old (mean = 18.4) Anxiety: 18–19 years old (mean = 18.2)	Controls: 42 female, 8 male Anxiety: 21 female, 4 male	Controls: 7 Asian, 4 African American, 2 MTOR, 2 UK/NR, 35 White Anxiety: 7 Asian, 2 African American, 3 MTOR, 13 White	3 moderate 23 severe
Study B	Controls: 15–17 years old (mean = 15.7) Anxiety: 15–17 years old (mean = 15.8)	Controls: 38 female, 44 male Anxiety: 19 female, 22 male	Controls: 15 Asian, 10 African American, 6 MTOR, 2 UK/NR, 49 White Anxiety: 7 Asian, 5 African American, 4 MTOR, 25 White	30 moderate 11 severe
Study C	Controls: 18–24 years old (mean = 18.4) Anxiety: 18–24 years old (mean = 18.2)	Controls: 26 female, 22 male Anxiety: 26 female, 22 male	Controls: 1 Asian, 3 African American, 10 MTOR, 1 UK/NR, 33 White Anxiety: 3 Asian, 2 African American, 6 MTOR, 1 UK/NR, 36 White	9 moderate 39 severe

*Note:* Studies A and B were matched two‐to‐one (each anxiety subject had two matched control subjects), while study C was matched one‐to‐one. The racial demographics available for assessment were Asian, African American, MTOR (More Than One Race), UK/NR (Unknown or Not Reported), and White.

### 2.2. Anxiety Symptom Scoring

Each of our originating studies evaluated anxiety symptoms with a different but well‐established method. Study A utilized the GAD‐7 scale. Study B evaluated anxiety using the Beck Anxiety Inventory (BAI) [34]. Anxiety subjects in study C were clinically diagnosed prior to inclusion and were evaluated with the State–Trait Anxiety Inventory (STAI). We utilized the trait anxiety score within the STAI to assess anxiety severity. Each of these assessments has high validity in assessing the severity of anxiety symptoms [35–38].

Based on the scoring interpretation provided by Spitzer et al. [39], we determined that GAD‐7 total scores were categorized as: 0–4, no anxiety; 5–9, mild anxiety; 6–10, moderate anxiety; 10–21, severe anxiety. BAI ratings were categorized as: 0–7, minimal anxiety; 8–15, mild anxiety; 16–25, moderate anxiety; 26–63, high anxiety. STAI scores, ranging between 20–80, were categorized as: 20–37, no/low anxiety; 38–44, moderate anxiety; 44–80, high anxiety [40]. Study A control and anxiety subjects had mean GAD‐7 scores of 0.55 (standard error of the mean [SEM]: 0.13) and 11.0 (SEM: 0.54), respectively. Study B control and anxiety subjects had mean BAI scores of 3.1 (SEM: 0.11) and 24.3 (SEM: 0.43), respectively. Study C control and anxiety subjects had mean STAI scores of 27.7 (SEM: 0.18) and 49.0 (SEM: 0.36), respectively. These anxiety cohorts were therefore in the high‐moderate or severe range for each originating study.

### 2.3. MRI Data Acquisition

All subjects were scanned at 3T using whole‐brain diffusion tensor imaging (DTI) and T1 (MPRAGE) protocols. All MRI scans were completed in single sessions. DTI data for studies A and C were collected at isotropic resolution (2 mm and 1.5 mm, respectively), while study B was collected at 0.86 mm x 0.86 mm x 3.0 mm. Studies A and B utilized 69 diffusion directions, while study C utilized 199 diffusion directions. Additional DTI parameters are available in the studies associated with the originating data [31–33]. We included study‐of‐origin as a covariate when quantifying compartment‐like volume to control for the influence of differing acquisition parameters. Additionally, we carried out whole‐brain segmentation using the Freesurfer utility *recon-all* (version 7.1.1), which standardizes T1 volumes to 1 mm isotropic resolution, regardless of the starting resolution.

### 2.4. DTI Preprocessing

We processed raw DTI volumes using FSL tools (version 6.0.7.1, https://pages.fmrib.ox.ac.uk/fsldocs-4c7e2c/). We performed skull stripping of DTI volumes using *bet2*. We corrected for eddy current‐induced distortions and motion artifacts using *eddy*. Studies A and B included a single anterior‐to‐posterior acquisition, while study C included both anterior‐to‐posterior and posterior‐to‐anterior acquisitions. We used *topup* to correct susceptibility‐induced distortion for study C. We fit diffusion tensors for each image using *dtifit*, generating a 3D fractional anisotropy (FA) image with the same resolution as the original DTI image. Finally, we generated diffusion parameter estimates and modeled crossing fibers within each voxel using *bedpostx* [41]. We transformed region‐of‐interest masks from Montreal Neurological Institute (MNI) standard space into each subject’s native DTI space using affine (*flirt*) and nonlinear (*fnirt*) registration tools. We performed visual inspections of the products of each DTI processing step.

### 2.5. Striatal Parcellation With Classification Targets Tractography (CTT)

**Figure 1:**
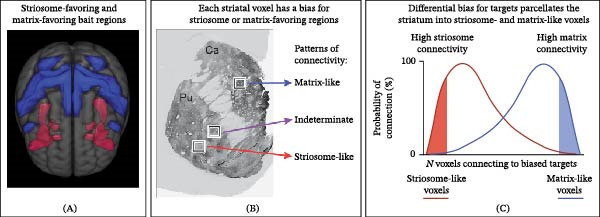
Biases in structural connectivity identified striosome‐like and matrix‐like voxels. Animal studies that utilized injected tract tracers identified regions with biased projections, either striosome‐favoring or matrix‐favoring (A), that we utilized as “bait” for connectivity‐based parcellation. In (B), tyrosine hydroxylase immunohistochemistry in human striatal tissue identified striosome (light stain) and matrix (high stain) in the caudate (Ca) and putamen (Pu). This immunohistochemistry is shown for illustration only; this study included only living subjects. The voxels of diffusion MRI sample this tissue in a rigid grid, independent of the location of the striosome and matrix. At random, MRI sampled voxels that included more matrix, more striosome, or a blend of the two (indeterminate). Striatal parcellation identifies those voxels with high bias toward one compartment (C), discarding the indeterminate and low‐bias voxels in the center of the distribution.

We performed probabilistic tractography in each subject’s native diffusion space. We first used CTT mode in the FSL tool *probtrackx2* to parcellate the striatum into matrix‐like and striosome‐like compartments, as described previously (Figure 1) [27]. This connectivity‐based parcellation method uses five striosome‐favoring regions and five matrix‐favoring regions, identified in prior studies that injected tract tracers in animals [27], as “bait” regions to identify striatal voxels with biased patterns of connectivity. We have previously utilized these 10 bait regions to parcellate the striatal compartments in different imaging datasets [12, 13, 18, 42, 43]. Our five matrix‐favoring regions included the caudodorsal part of the inferior frontal gyrus pars opercularis, primary motor cortex, supplementary motor area, primary somatosensory cortex, and superior parietal cortex. Our five striosome‐favoring regions included the posterior orbitofrontal, anterior insula, basolateral amygdala, basal operculum, and the posterior temporal fusiform cortex. All 10 standard space bait regions are provided in the Supplemental Materials. We summed all striosome‐favoring, or all matrix‐favoring regions to generate composite masks. Each bait region has biased projections toward one compartment, but that bias may not be uniform throughout the region. Additionally, compartment‐specific bias is somatotopically organized and, for any given bait region, is limited to one part of the striatum [44]. Composite masks help account for these within‐region sources of variance.

Our striatal seed mask included the caudate and putamen and excluded the nucleus accumbens and posterior half of the caudate tail, as this region of the caudate led to registration errors and partial volume effects due to its small size in coronal sections [27]. CTT used each striatal voxel as a seed, quantifying the structural connectivity of that voxel with the summed striosome‐favoring and matrix‐favoring bait masks. CTT utilized the following tractography parameters: curvature threshold = 0.2; step length = 0.5 mm; 2000 steps per sample; 5000 streamlines per seed voxel. The result of this tractography was two overlapping striatal probability maps that demonstrated striosome‐like or matrix‐like structural connectivity. The ratio between these probability maps defined the bias toward striosome‐favoring or matrix‐favoring bait regions and thus defined each voxel’s identity as striosome‐like, matrix‐like, or indeterminate. Because the resolutions of our diffusion voxels were larger than the upper limit for the diameter of human striosome tubules, all striosome‐like voxels and many matrix‐like voxels included tissue “contaminant” from the opposing compartment. This resulted in many striatal voxels with indeterminate or weak bias toward one compartment. Therefore, we selected the most biased voxels to represent the two compartments in subsequent rounds of tractography. The output of probabilistic tractography may be distorted if target masks have unequal volumes. To minimize this source of variability, we ensured that striosome‐like and matrix‐like masks had equal volume in each hemisphere. To generate these equal‐volume striosome‐like and matrix‐like masks, we started at the upper limit of compartment‐like bias (*p* = 1.0) and iteratively reduced the probability threshold until we reached the target voxel count, set to match the uppermost 1.5 standard deviations of striatal volume [27]. As our three cohorts had differing DTI resolutions, each cohort utilized a different target voxel number for their compartment‐like masks, but these voxels comprised the same fraction of striatal volume in each cohort. As these compartment‐like masks were inferential and probabilistic, we labeled these voxels as “striosome‐like” and “matrix‐like” to remind readers that this method is not a direct identification of striosome or matrix tissue. We utilized these equal‐volume (striosome volume = matrix volume) masks for measures of diffusivity, location, and for subsequent rounds of tractography. All code used to generate these striatal parcellations, along with the bait, seed, and exclusion masks used in striatal parcellation, can be accessed here github.com/jeff‐waugh/Striatal‐Connectivity‐based‐Parcellation.

### 2.6. Compartment‐Like Volume and Diffusivity

We measured the volume (mm^3^) of voxels within the striosome‐like and matrix‐like probability distributions (*p* = 0.55–1.0 for each distribution) for each subject, separately in each hemisphere. We also extracted the volume within the indeterminate range (0.45–0.55, not part of either compartment‐like distribution). We measured striatal volume in diffusion‐space (the seed mask utilized for striatal parcellation) and normalized for head size using the estimated total intracranial volume (eTIV) derived from *recon-all* parcellation of each subject’s T1 image. We measured FA and radial diffusivity (RD) within striosome‐like and matrix‐like masks (the uppermost 1.5 standard deviation volumes for each probability distribution) and also within the whole striatum. For both volume and diffusion metrics, the variance between studies was greater than the variance within studies. Therefore, we mean‐centered FA and RD within each study and expressed volume, FA, and RD as a percentage above or below the normalized mean.

### 2.7. Intrastriate Location

For each subject and hemisphere, we measured the Cartesian position of each voxel in our highly biased matrix‐like and striosome‐like masks, comparing each voxel’s location to the centroid of its nucleus of origin (caudate or putamen). We assessed the location of striosome‐like and matrix‐like voxels in each plane (*x*, *y*, and *z*) and also calculated the root–mean–square distance from the centroid.

### 2.8. Voxelwise Analysis of Compartment‐Like Bias With *Randomize*

Compartment‐like bias may vary in amplitude, location, or complex interactions of amplitude and location. A voxelwise assessment of compartment‐like bias is key to identifying both the valence of change and the location at which anxiety and control subjects differ. Striosome‐like and matrix‐like probability biases always sum to one at each striatal voxel, so voxelwise assessments of matrix‐like bias are approximately equal to the inverse of those for striosome‐like bias—it was sufficient to assess only matrix‐like bias. We used the nonparametric permutation inference tool *randomise* to compare matrix‐like bias between anxiety and control subjects at each striatal voxel. We completed 5000 permutations, masked by the striatal seed mask, utilized variance smoothing (2 mm) and threshold‐free cluster enhancement, and corrected for multiple comparisons using the family‐wise error (FWE) method. We combined each subject’s left and right hemispheres into a single volume to reduce the number of *randomise* comparisons. For voxels that significantly differed between anxiety and control subjects, we dilated this significant volume by 1 mm in each plane to smooth irregularities. We used this *randomise*‐derived volume, which we termed the rostral putaminal zone (RPZ), as the target for subsequent rounds of tractography.

### 2.9. Assessment of Striosome‐Like Volume by Coronal Plane

We extracted striosome‐like volume in 2 mm coronal planes that spanned the rostral‐caudal extent of our striatal mask (+26 to −33 mm, (MNI coordinates). The left and right hemisphere striatal masks were offset in the MNI template brain by 1 mm, so we shifted the right hemisphere measures rostrally by one slice. We extracted striosome‐like volume separately in caudate and putamen since their volumes overlap in the coronal plane.

### 2.10. Analyses of Compartment‐Like Bias Within and Outside the RPZ

Areas without a significant difference in voxelwise comparisons may have no between‐group amplitude differences, or there may be amplitude differences that vary in location within the group and therefore go uncounted. This is especially relevant for striatal compartment differences since striosome and matrix are distributed uniquely within each individual. We defined a striatal mask based on areas where anxiety and control subjects significantly differed in the amplitude of compartment‐like bias, termed the RPZ (see Methods 2.8). We assessed connectivity within and outside the RPZ in two ways; first, we extracted mean connectivity with striosome‐favoring and matrix‐favoring bait regions, within and outside the RPZ; second, we seeded tractography from striosome‐favoring or matrix‐favoring bait regions and targeted either the RPZ or the striatum excluding the RPZ (waypoint: striatum_minus_RPZ; exclusion: RPZ). We used the same tractography parameters described above, except for utilizing streamline instead of CTT. For every subject we characterized the ratio of counts that reached RPZ:non‐RPZ striatum.

### 2.11. Quantified Connectivity Between Bait Regions and Striatal Compartments

Shifts in the strength of compartment‐like connectivity can be driven by abnormalities in striosome‐favoring bait regions, matrix‐favoring bait regions, or both. Therefore, we set out to quantify compartment‐specific bias among our bait regions. These tests required that we remap striosome‐like and matrix‐like connectivity—one cannot use a bait region to define compartment‐like bias within the striatum and then accurately quantify connectivity with that bait region. For example, if one defines striosome‐like voxels as “those with high bias toward the amygdala” then connectivity between the amygdala and striosome‐like voxels will always be high. Instead, we performed a series of *N* − 1 (“leave one out”) parcellations, one for each of our 10 bait regions [13, 27, 43]. Briefly, we performed CTT as described above but utilized nine instead of 10 bait regions (four favoring one compartment, five favoring the other compartment). We performed 10 rounds of *N* − 1 CTT, rotating the left‐out region among our 10 bait regions. Next, we selected highly biased striosome‐like and matrix‐like striatal masks from each *N*−1 parcellation—new striosome‐like and matrix‐like masks whose parcellations were not influenced by the left‐out region.

Having defined new compartment‐like masks that were not influenced by a specific bait region, we could then measure connectivity between the left‐out bait region (seed) and these *N*−1 compartment‐like masks (targets) in subsequent rounds of CTT, one for each of the 10 bait regions. We quantified the volume of each bait region that was biased (*p* ≥ 0.55) toward its target compartment (matrix‐favoring bait regions to matrix‐like voxels, striosome‐favoring bait regions to striosome‐like voxels). We normalized the biased volume within each bait region by mean‐centering across all subjects (anxiety and control) for that bait region. This was essential to account for the large volume differences between bait regions (e.g., the primary motor cortex is 38‐fold larger than the basolateral amygdala). We then summed the mean‐centered volume measures from our five striosome‐favoring or five matrix‐favoring regions to provide a composite assessment of compartment‐specific bias within our bait regions. We wished to learn which bait regions contributed to shifts in compartment‐like striatal volume. Therefore, we performed a post hoc analysis of each bait region individually, comparing anxiety and control cohorts on the volume of that bait region (mean‐centered) that was biased toward striosome‐like or matrix‐like voxels.

### 2.12. Influence of Bait Regions Within the RPZ

In order to determine the influence of bait regions on compartment‐like volume in the RPZ, we performed CTT using nine bait regions, leaving one out as our test region. We then subtracted that *N*−1 distribution from the original CTT that utilized all 10 bait regions for parcellation. This isolated the contributions of the left‐out region to compartment‐like connectivity. We completed this process for each of our 10 bait regions and then extracted mean connectivity within the RPZ for each “difference volume.” This allowed us to assess the contribution of each bait region on compartment‐like bias. We extracted the mean connectivity within the RPZ for each of bait regions, resulting in a mean intensity value for each striosome‐favoring or matrix‐favoring bait region for each subject and hemisphere. We averaged the mean intensity values for these *N* − 1 regions within anxiety and control cohorts and compared mean intensity between our two cohorts.

### 2.13. Whole‐Brain Assessment of White Matter Integrity

We aimed to identify any shared white matter structural abnormalities in anxiety subjects that might distort striatal parcellation or quantitative assessments of corticostriate tractography. Therefore, we carried out voxelwise statistical analysis of the whole brain white matter, comparing FA between anxiety and control subjects, using Tract‐Based Spatial Statistics (TBSS) [45] in FSL. First, we registered all subjects’ FA volumes to a study‐specific template, the nonlinear registration tool fnirt. Next, we generated a mean FA image that included all subjects (anxiety and controls). We thinned this averaged image volume to create a mean FA skeleton, which represented the core of all white matter tracts shared within the group. We projected each subject’s aligned FA data onto this skeleton and performed voxelwise between‐group comparisons of FA. We corrected for FWE due to multiple comparisons within TBSS.

### 2.14. Statistical Tests

Nonparametric, voxelwise comparisons of compartment‐like connectivity bias were carried out using *randomise*, as detailed in Methods 2.8. We assessed whole striatal volume, compartment‐like volume, diffusivity, intrastriate location, biased volume within bait regions, and region‐specific contribution within the RPZ using a series of two‐tailed unpaired *t*‐tests that compared group means between our anxiety and control cohorts. We tested whether mean connectivity with the RPZ and non‐RPZ striatum followed the same pattern of bias seen in whole‐striatum comparisons using one‐tailed unpaired *t*‐tests, comparing anxiety and control cohorts. We assessed striosome‐like connectivity within the RPZ with ANOVA, covarying by anxiety severity, clustered into categories using the cutoffs listed in Methods 2.2, and by study of origin as a nuisance factor. We used a one sample *t*‐test to assess the mean striosome:matrix volume ratio relative to the previously established striosome:matrix ratio from histology. We corrected for false discovery rate (FDR) within each family of tests using the method of Benjamini and Hochberg [46].

## 3. Results

### 3.1. Global Assessments: Whole‐Brain White Matter and Striatum

Our combined cohort included 115 anxiety subjects matched with 180 healthy controls (Table 1). White matter integrity (as assessed by FA) did not differ between anxiety and control subjects at any white matter voxel. Striatal volume (normalized by intracranial volume) did not differ between anxiety and control subjects (0.58 vs. 0.58; *p* = 0.47). Whole‐striatum FA and RD did not differ between anxiety and control subjects (FA: 0.98% smaller in anxiety, *p* = 0.074; RD: 0.25% smaller in anxiety, *p* = 0.71). Striatal volume, FA, and RD also did not differ between anxiety and controls in any of the three studies that comprised our combined cohort (*p*‐values for striatal volume (0.43, 0.25, 0.23), for FA (0.22, 0.55, 0.93), and for RD (0.56, 0.39, 0.11).

### 3.2. Compartment‐Like Voxels: Volume, Diffusivity, and Location

**Figure 2:**
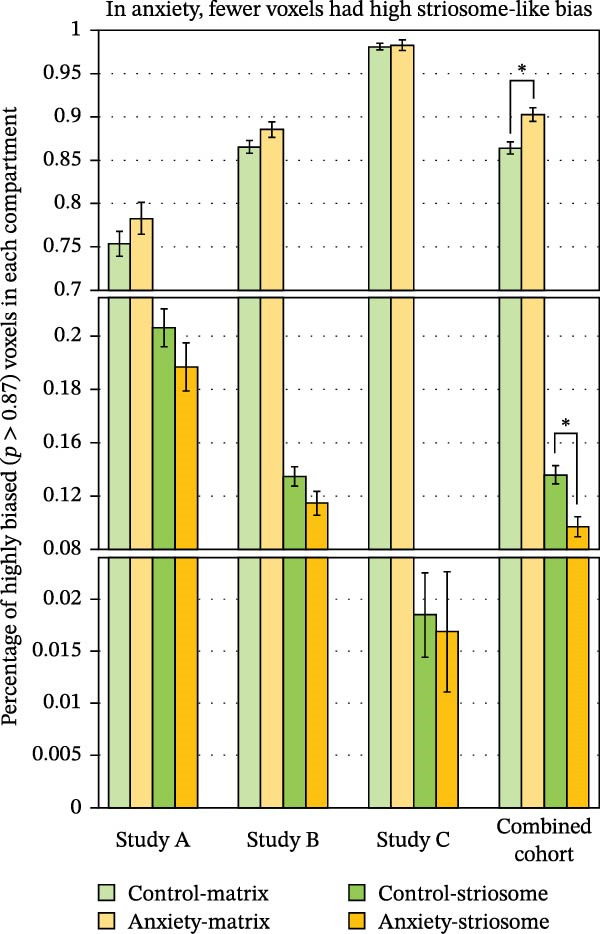
In adolescents and young adults with anxiety (*N* = 115), highly biased voxels (connection probability ≥ 0.87) were shifted from striosome‐like to matrix‐like connectivity, relative to matched healthy controls (*N* = 180). This shift was evident in each of the included studies (solid orange bars are lower than solid green bars in A, B, and C), though anxiety‐vs.‐control differences were significant only in the combined cohort. Error bars represent the standard error of the mean.  ^∗^ *p* = 2.6 × 10^−4^.

We previously established that the volume of highly biased (*p* ≥ 0.87) compartment‐like voxels can identify group‐level differences in striosome‐like and matrix‐like structural connectivity [12, 13, 18, 27, 47]. In the complete dataset (anxiety plus controls), the relative volume of highly biased voxels was 12.1% striosome‐like and 87.9% matrix‐like (SEM 0.52%; one‐sample *t*‐test, relative to the ratio expected from histology, *p* = 0.42), closely approximating the expected striosome:matrix ratio of 15:85 established in human tissue [48, 49]. Control subjects hewed closely to this ratio (13.6:86.4), while anxiety subjects differed substantially from the expected ratio (9.7:90.3; anxiety vs. histologic standard, *p* = 4.8 × 10^−11^). Striosome‐like volume was reduced by 28.6% in anxiety subjects relative to matched controls (9.7% vs. 13.6%; *p* = 2.6 × 10^−4^, Figure 2). Correspondingly, the matrix‐like compartment was increased by 4.5% in anxiety subjects (90.3% vs. 86.4%; *p* = 2.6 × 10^−4^). This shift of volume from striosome‐like to matrix‐like was consistent in each of the studies that made up our experimental cohort, though this difference was significant only in the combined dataset.

Shifts in compartment‐like volume may arise from changes in the composition of striosome‐like, matrix‐like, or both compartments. We investigated whether changes in diffusion metrics correlated with these shifts in compartment‐like volume. In the complete dataset (anxiety plus controls) normalized FA was 18.9% higher in striosome‐like voxels than in matrix‐like voxels (1.2 vs. 1.0; *p* = 2.1 × 10^−30^). Similarly, normalized RD was 5.4% higher in striosome‐like voxels than in matrix‐like voxels (1.0 vs. 0.96; *p* = 1.1 × 10^−10^). These differences suggest that tissue included in striosome‐like voxels (for both anxiety and control subjects) differs in composition and/or organization from that included in matrix‐like voxels, as we previously demonstrated [27]. Differences between anxiety and control subjects, in contrast, only trended toward significance for normalized FA and RD and suggested that anxiety subjects may have had less, rather than more, tissue disruption in striosome‐like voxels. In anxiety subjects, striosome‐like FA was increased (+4.8%, *p* = 0.083) and striosome‐like RD was decreased (−3.0%, *p* = 0.065) relative to matched controls. Anxiety‐vs.‐control differences in FA and RD were substantially smaller in matrix‐like voxels (FA, +1.5% in anxiety, *p* = 0.52; RD, −1.9% in anxiety, *p* = 0.077).

We investigated if compartment‐like volume differences between anxiety and control subjects localized to different parts of the striatum. In histology, the striosomes is generally enriched in the rostral, medial, and ventral striatum, though one can identify striosome at lower abundance throughout the striatum. The matrix, conversely, makes up a larger proportion of the caudal, lateral, and dorsal striatum. In both anxiety and control subjects, striosome‐like and matrix‐like voxels matched the spatial distribution demonstrated in histology (for *x*‐, *y*‐, and *z*‐planes, all *p* ≤ 1.3 × 10^−39^). However, this intercompartmental segregation was larger in the putamen of anxiety subjects bilaterally: striosome‐like voxels were shifted more caudal (1.0 mm, *p* = 4.8 × 10^−41^), lateral (1.3 mm, *p* = 3.3 × 10^−131^), and dorsal (1.3 mm, *p* = 2.3 × 10^−8^), relative to controls. Putaminal matrix‐like voxels were shifted slightly toward striosome‐like positions: more rostral (0.53 mm, *p* = 7.7 × 10^−19^), lateral (0.05 mm, NS), and ventral (1.1 mm, *p* = 3.6 × 10^−9^) in anxiety subjects.

Matrix‐like voxels in the caudate in anxiety subjects were shifted more caudal (2.6 mm, *p* = 1.9 × 10^−104^), ventral (0.26 mm, 5.5 × 10^−11^), and lateral (0.8 mm, *p* = 1.6 × 10^−55^). Matrix‐like and striosome‐like voxels in anxiety subjects were dispersed more than in controls, in both the caudate and putamen and in both hemispheres. The root–mean–square distances (centroid‐to‐compartment‐like voxel) in anxiety subjects were significantly larger for both striosome‐like voxels (8.2 vs. 6.6 mm, anxiety vs. control; *p* = 2 × 10^−248^) and matrix‐like voxels (11.4 vs. 5.3 mm, anxiety vs. control; *p* ≤ 1 × 10^−248^).

### 3.3. Localization of Abnormal Striosome‐Like Connectivity

**Figure 3:**
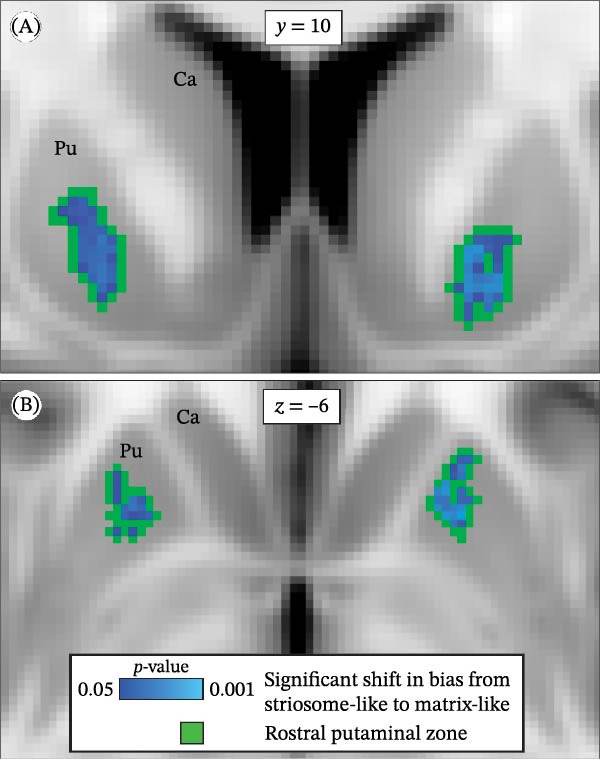
In subjects with anxiety, voxels with significant shifts from striosome‐like to matrix‐like connectivity were limited to a bilateral zone in the rostral putamen. We compared connectivity bias in anxiety subjects and matched controls using *randomise* with threshold‐free cluster enhancement, seen in the coronal (A) and axial (B) planes. While left‐ and right‐hemisphere rounds of tractography were executed independently, both identified the same rostral putaminal zone. The mean striosome‐like bias in the rostral putaminal zone was reduced by 14.1% in anxiety subjects. No other area in caudate or putamen had significant shifts in bias. Blue‐light blue voxels: significant shift (*p* < 0.05, FWE‐corrected for multiple comparisons) in compartment‐like bias. Green voxels: the rostral putaminal zone (RPZ) that included significant voxels from *randomise*, expanded by one voxel in each plane. Ca = caudate. Pu = putamen. Coordinates follow Montreal Neurological Institute (MNI) convention.

We assessed compartment bias at a voxelwise level using the FSL tool *randomise* to identify the striatal locations where compartment‐like structural connectivity was abnormal in anxiety (Figure 3). Though we parcellated the striatal compartments independently in each hemisphere, both hemispheres had shifts from striosome‐like toward matrix‐like connectivity in a highly similar location, the RPZ. The RPZ had similar volume (left: 148 mm^3^; right: 157 mm^3^) and location bilaterally (interhemispheric differences in center of gravity, *y*‐plane: 0.44 mm; *z*‐plane: 3.7 mm). No other parts of the striatum had significant shifts in bias. Note that the isolated nature of this striosome‐to‐matrix shift does not mean that no other shifts in bias occurred in other parts of the striatum, only that other shifts, if present, were spatially heterogeneous.

**Figure 4:**
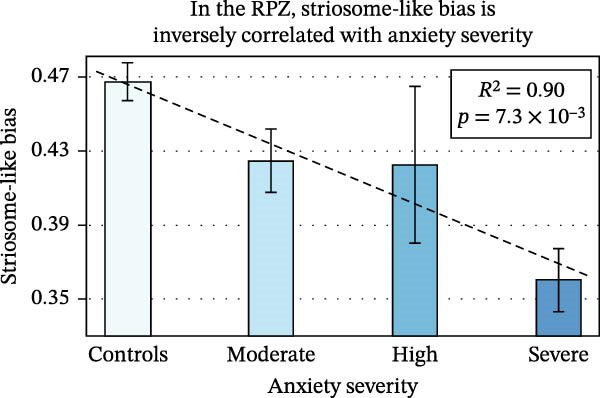
In the rostral putaminal zone (RPZ), striosome‐like connectivity was inversely related to the severity of anxiety. For each study we divided subjects into severe, high, moderate, or no anxiety based on standardized cutoffs for each anxiety battery. *R* ^2^ and *p*‐value determined by ANOVA.

Mean striosome‐like connection probability within the RPZ was 14.1% lower in anxiety subjects than in controls (0.40 vs. 0.47; *p* = 3.8 × 10^−5^). In contrast, the mean striosome‐like connection probability in the rest of the striatum (excluding the RPZ) did not differ between anxiety and control subjects (0.276 vs. 0.283, respectively; *p* = 0.30). Striosome‐like connectivity was inversely correlated with the severity of anxiety (Figure 4): control subjects, 0.467; moderate anxiety, 0.425; high anxiety, 0.422; severe anxiety, 0.360; *p* = 7.3 × 10^−3^). Striosome‐like connectivity in anxiety subjects was decreased more in the left RPZ than in the right (−16.0% vs. −12.2%, respectively; *p* = 8.5 × 10^−3^). Matrix‐like connectivity within the RPZ was 12.3% higher in anxiety subjects (0.60 vs. 0.53, respectively; *p* = 3.8 × 10^−5^). This shift from striosome‐like to matrix‐like connectivity in the RPZ is congruent with the previously noted location shift, with matrix‐like voxels displaced rostrally, medially, and ventrally, (closer to the RPZ) while striosome‐like voxels were dispersed caudally, laterally, and dorsally in the putamen (further from the RPZ).

**Figure 5:**
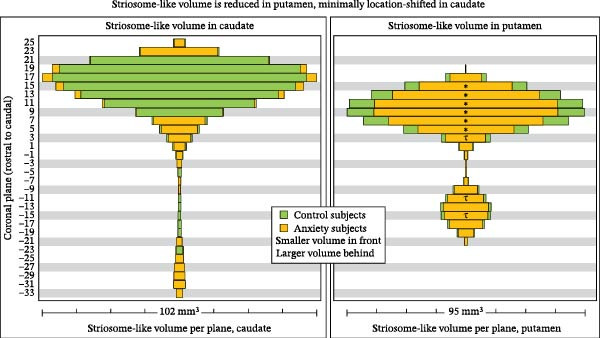
We extracted striosome‐like volume in each coronal plane spanning the striatum (rostral‐to‐caudal = top‐to‐bottom) in control subjects (green) and anxiety subjects (orange). For every plane, the smaller‐volume cohort is placed in front to illustrate the volume difference between cohorts. While striosome‐like volume was reduced throughout the putamen (right panel–orange is always in front), volume in the caudate was shifted from lower‐density to higher‐density locations but not reduced (b).  ^∗^ *p* < 0.014, the FDR‐corrected significance threshold; τ, *p* < 0.05, trend toward significance. Coordinates follow MNI convention.

To identify potential shifts in bias that were spatially dispersed and thus not evident with voxelwise testing, we measured striosome‐like volume (*p* = 0.55–1) in 2 mm‐thick coronal planes, separately in the caudate and putamen. Striosome‐like volume was reduced in anxiety subjects throughout the putamen, with the largest reductions in planes that included the RPZ (Figure 5; planes 5–18; total reduction: 21.2%; range of reduction across all planes: 2.8–51.8%). In contrast, striosome‐like volume in the caudate was shifted from higher‐density to lower‐density planes but was not reduced (minimum *p*‐value for caudate planes, *p* = 0.20; total striosome‐like volume increased by 2.9%).

### 3.4. The Contribution of Individual Bait Regions to Compartment‐Like Bias

**Figure 6:**
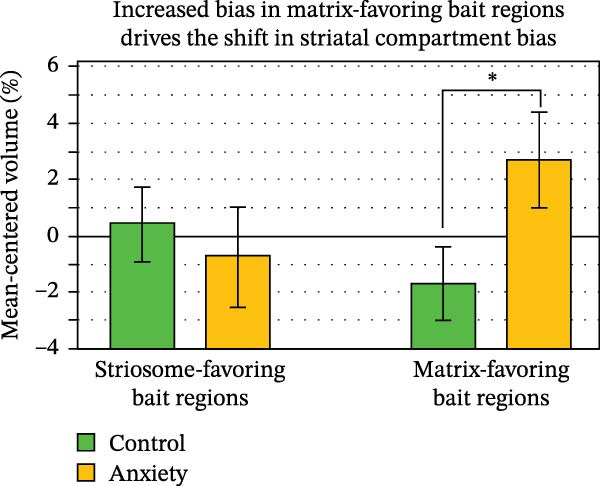
The shift in striatal bias toward matrix‐like connectivity is driven by increased structural connectivity within the matrix‐favoring bait regions. Anxiety subjects were slightly decreased in striosome‐like volume, but not significantly different. Error bars represent the standard error of the mean.  ^∗^ *p* = 0.021.

**Figure 7:**
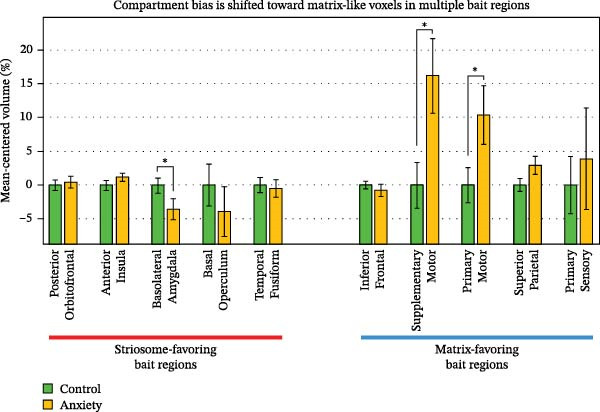
Within each bait region, we assessed the volume that was biased toward either striosome‐like or matrix‐like voxels. In anxiety subjects, bias was shifted from striosome‐like to matrix‐like in three bait regions: basolateral amygdala, supplementary motor, and primary motor. Volume is normalized to the control cohort for each bait region. Error bars represent the standard error of the mean.  ^∗^ *p* < 0.05.

Shifts in the volume and location of compartment‐like voxels could result from abnormalities in our bait regions, in the striatum, or both. In rounds of tractography seeded by either striosome‐favoring or matrix‐favoring bait regions, we quantified structural connectivity (number of completed streamlines) when tractography targeted the RPZ, or the striatum excluding the RPZ. For streamlines seeded by matrix‐favoring regions, the RPZ:non‐RPZ ratio was increased by 25.6% in anxiety subjects, relative to controls (0.0048 vs. 0.0038, respectively; *p* = 0.019). In contrast, the RPZ:non‐RPZ ratio did not differ for streamlines seeded by striosome‐favoring bait regions (anxiety, 0.038; control, 0.039; *p* = 0.43). The ratio of streamlines seeded by matrix‐favoring:striosome‐favoring regions (MF:SF) suggests a similar spatially‐restricted abnormality in structural connectivity. In the RPZ, the MF:SF was 80.0% larger in anxiety subjects than in controls, trending toward significance (4.9, SEM + 1.1 vs. 2.7, SEM + 0.33, respectively; *p* = 0.031). In the non‐RPZ striatum, the MF:SF ratio was not different in anxiety and control subjects (11.9, SEM + 0.94 vs. 13.6, SEM + 0.86, respectively; *p* = 0.086). Notably, the RPZ included only 4.0% of the striatum. Anxiety‐related abnormalities in the non‐RPZ striatum may differ from those we described in the RPZ. We then assessed each bait region’s contribution to compartment‐like bias throughout the striatum. We performed *N*−1 (“leave‐one‐out”) striatal parcellation for each bait region and then mapped connectivity between the left‐out region and the *N*−1 parcellated compartment‐like voxels (seeds: each bait region in successive rounds of tractography; targets: compartment‐like voxels throughout the striatum). We summed these maps for all striosome‐favoring, and for all matrix‐favoring, bait regions. The volume of matrix‐favoring voxels within matrix‐favoring bait regions was significantly elevated in anxiety subjects (+4.9%; *p* = 0.021, Figure 6). In contrast, striosome‐favoring volume within the striosome‐favoring bait regions was only slightly decreased (−0.78%; NS). We performed a post hoc analysis of each bait region to determine which were the drivers of these shifts in bias (Figure 7). In anxiety subjects, matrix‐favoring volume was increased in the supplementary motor (+16.2%, *p* = 8.5 × 10^−3^) and primary motor cortices (+10.4%, *p* = 0.029). Striosome‐favoring volume was decreased in the basolateral amygdala (−3.6%, *p* = 0.046). No other bait regions were significantly different between anxiety and control subjects. This broad pattern of modestly shifted bias, from striosome‐favoring to matrix‐favoring, within our bait regions was one driver of the shift from striosome‐like to matrix‐like striatal volume in anxiety subjects.

Next, we assessed the contribution of each bait region within the RPZ. Striosome‐like bias contributed by the basal operculum was significantly decreased in anxiety subjects (−28.8%, *p* = 6.9 × 10^−6^) and trended toward a decrease for the basolateral amygdala (−19.5%, *p* = 0.037).

### 3.5. Histogram Assessment of Compartment‐Like Bias

Diffusion imaging samples striatal tissue in predictable patterns based on the dimensions of striatal voxels relative to the maximum diameter of striosome tubules. Therefore, changes to the distribution of compartment‐like voxels can provide inferences regarding the tissue‐level characteristics of the striatal compartments. We performed a histogram analysis of the full distribution probability map (*p*_range_ = 1.0–0.0, Figure 8, limited to the putamen since volume changes in the caudate were insignificant). Note that striosome‐like and matrix‐like distributions are mirrored copies; since at each voxel the probability of connectivity to matrix‐favoring regions and striosome‐favoring regions always sums to one, this approach allowed us to assess both compartments in one analysis. In anxiety subjects, striosome‐like connectivity was shifted from high‐bias to low‐bias striosome‐like connectivity and into a larger volume of indeterminate voxels (*p* = 0.45–0.55; +15.2% in anxiety; *p* = 2.8 × 10^−9^).

Figure 8Histograms of the bias in putaminal structural connectivity demonstrate that in anxiety subjects (orange), high‐bias striosome‐like volume (mm^3^) is shifted into low‐bias striosome‐like volume and matrix‐like volume, relative to matched healthy controls (green). The same data are presented as raw volume (A) and as normalized volume (anxiety normalized to controls) for easier visualization (B). Each bin includes the volume in a 0.02‐unit range spanning the full probability distribution (*p* = 1.0–0), with ranges denoted for striosome‐like bias (1.0–0.55, yellow–red), indeterminate (0.55–0.45), and matrix‐like bias (0.45–0, blue‐light blue).  ^∗^ *p* < 0.014, the FDR‐corrected significance threshold; *τ*, *p* < 0.05, trend toward significance.

**Figure:**
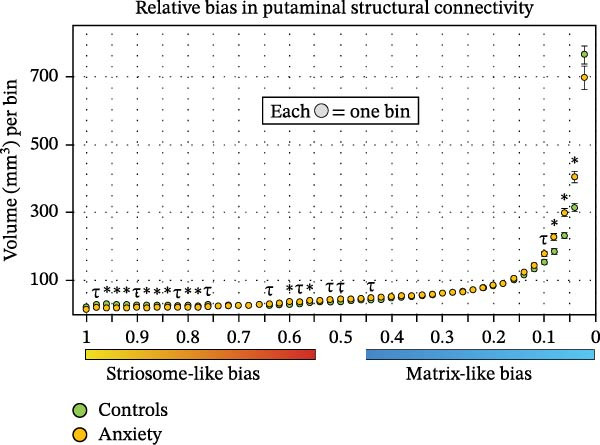
(A)

**Figure:**
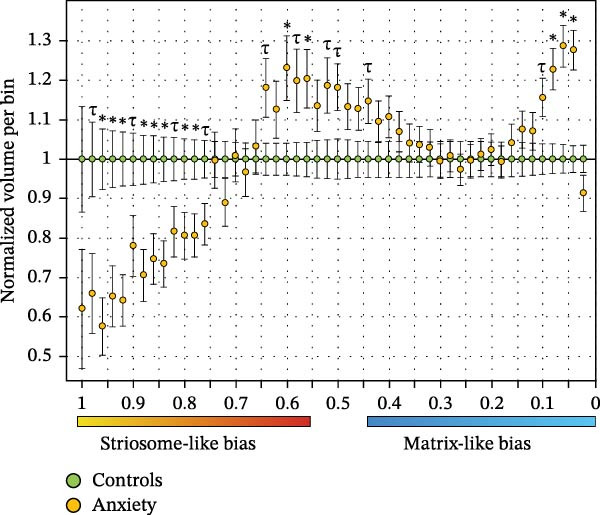
(B)

Notably, this shift did not lead to a meaningful change in total striosome‐like volume, but rather a change in which bins sampled striosome‐like voxels: the sum of all striosome‐like bins was only 2.5 mm^3^ smaller (−0.20%) in anxiety subjects than in controls – the volume reduction demonstrated in Figures 1−3 primarily impacted high‐bias voxels. Matrix‐like volume was significantly expanded in high‐bias bins (*p* = 0.92–0.96; *p* < 4.0 × 10^−4^ for each of these bins). However, matrix‐like volume was reduced in the highest‐bias matrix‐like voxels, though not significantly so (−8.9%; *p* = 0.12) – fewer matrix‐like voxels sampled no striosome tissue (Figure 8A). This pattern is consistent with a putative reduction in the packing density of the striosome, with striosome‐like connectivity infiltrating more voxels that, in control subjects, would sample exclusively or predominantly matrix‐like connectivity. This shift in bias in anxiety subjects was highly similar in female and male subjects (data not shown).

## 4. Discussion

The histologic features of the striatal compartments—their relative abundance, compartment‐specific biases in connectivity, and intrastriatal location [27, 50, 51]—are remarkably consistent throughout the mammalian lineage, suggesting that survival‐promoting striatal functions depend on the precise arrangement of striosome and matrix [12, 13, 18, 27, 50]. Broad differences in electrophysiology, pharmacology, and connectivity suggest that the compartments have separable functions. Striosome and matrix SPNs have distinct thresholds for intrinsic excitability [52] and opposing responses to striatal dopamine release [53]. Striosomal, but not matrix, afferents directly inhibit nigral dopaminergic projection neurons [52, 54]. Each compartment is embedded in distinct cortico–striato–thalamo–cortical loops [12, 18] and influences different functional networks [13, 21, 52]. Afferent and efferent projections are biased toward one compartment for >80% of the human diencephalon and telencephalon [27], suggesting that the majority of the brain’s functions are influenced by these compartment‐specific regulatory schemas.

In experimental animals, anxiety appears to also follow this divided function model. Striosomal activity encodes value‐based learning and correlates with task engagement and motivation [55], but this type of learning is disrupted by chronic stress [56]. In macaques, experimental activation of striosome‐projecting cortical regions induces anxiety‐like behaviors (pessimistic decision‐making, location‐avoidance) [57]. Similarly, in Huntington disease patients with significant anxiety the striosome suffered greater atrophy relative to Huntington patients without mood disorders [15]. Patients with Parkinson’s disease and high anxiety had diminished striatal dopamine transporter (DAT) availability in the rostral putamen [58]. DAT is striosome‐enriched, suggesting that the striosome is diminished in the rostral putamen is Parkinson’s patients with anxiety. Likewise, decreased functional connectivity between the putamen and orbitofrontal gyrus, a strongly striosome‐biased region [27], correlated with increased anxiety in Parkinson’s disease [59]. We recently demonstrated patients with Parkinson’s disease have a shift from striosome‐like to matrix‐like connectivity in the putamen, and the amplitude of that shift correlates with the severity of motor symptoms [42]. Whether shifts in the striosome‐matrix ratio in Parkinson disease correlate with anxiety is yet to be explored. However, it appears that abnormal striosome function could plausibly produce susceptibility to anxiety in neurodegenerative disorders.

We found that striosome‐like voxels were reduced in number and shifted in location in human anxiety, potentially establishing a mechanism for some anxiety disorders. The area of greatest abnormality in anxiety, the RPZ, was highly similar in size and location in the two hemispheres, though we parcellated left and right striata independently. The reduction in striosome‐like connectivity in the RPZ scaled inversely with anxiety severity—subjects with larger reductions in striosome‐like connectivity had higher scores on standardized measures of anxiety. The RPZ comprised only 4% of striatal volume, suggesting a specific focus for future investigations and, potentially, for targeted therapies. Importantly, anxiety and control subjects did not differ in the microstructural integrity of the white matter or the striatum, suggesting that the gray matter abnormalities we identified did not result from shared patterns of injury or abnormal development of the white matter. Instead, some forms of early‐onset anxiety disorder may result from developmental abnormalities of the striosome or its corticostriate connections. Intriguingly, we recently identified a striatal compartment abnormality in young people with major depressive disorder [60]. While the compartment‐like volume abnormalities in depression are different than those we identified here, both disorders had compartment‐specific abnormalities in the rostral putamen.

It is unknown how the functions of the striosome in caudate and putamen differ. However, their histochemical differences in humans—striosomal SPNs in the putamen cluster into larger, less abundant tubules with different immunohistochemical staining profiles than those in caudate [50]—suggest that internuclear differences in function arise from distinct developmental trajectories in the caudate and putamen. Indeed, our finding that striosome‐like voxels were reduced in volume in the putamen but shifted in location in the caudate underscores that different developmental abnormalities may be at play in each striatal nucleus. Future histological assessment of human striatal tissue from individuals with anxiety disorders is essential to confirm these findings and investigate the architecture of the human striosome, a feature that was beyond the resolution of our imaging method.

Our findings are based on probabilistic tractography, a technique with anatomic limitations. The resolution of our diffusion voxels was larger than the maximum diameter of striosome tubules, guaranteeing that our striosome‐like voxels also included a minority contribution from matrix tissue. Tractography cannot distinguish the directionality of connections and is blind to synapses, potentially leading to false positive streamlines or failure to resolve small‐volume streamline bundles [61]. Though we validated our probabilistic methods with prior findings in animal and human histology [12, 18, 27], compartment‐specific projections remain uncharacterized for many brain regions. Another limitation of our findings is that our cohorts are derived from three studies with different diffusion imaging protocols. Though we directly matched anxiety and control subjects within each study, the possibility remains that these differences in acquisition parameters distorted our findings. Similarly, each of our three originating studies quantified symptoms with a different anxiety inventory. We utilized published thresholds for severity for each anxiety test, eliminating subjects with mild anxiety as a buffer between anxiety and control cohorts. However, these differences in anxiety inventory may have led to nonuniform criteria for eliminating subjects.

Discovering the precise location and nature of neuroanatomic differences in individuals with anxiety is essential for understanding why these disorders occur. We identified a localized pattern of striosomal abnormalities in individuals with anxiety. However, histochemical characterization of the compartments in human tissue is necessary to understand the changes in striosome architecture that may underpin these abnormalities. Likewise, assessment of the functional connectivity networks that couple with the RPZ may provide a mechanism through which decreased striosomal function in the RPZ could produce the clinical phenotypes of anxiety. Future neuroimaging efforts to characterize the striatal compartments may reveal the sites where neuroanatomic abnormalities are shared among different forms of anxiety, versus compartment‐specific abnormalities that are unique to specific subtypes of anxiety. Noninvasive treatments for mood disorders, such as transcranial magnetic stimulation (TMS), are substantially improved by adjusting targets for individual differences in neuroanatomy and in functional networks [62]. TMS is utilized for anxiety [63] at much lower rates than for depression. We propose that individualizing TMS targets based on connectivity with the striosome, within the rostral putamen, may boost the efficacy of noninvasive brain stimulation for anxiety disorders. Finally, the anatomic precision of our findings suggests that structural brain injury to the rostral putamen, and in particular, to the striosome in the RPZ, may predispose to anxiety disorders. For example, 24% of survivors of cardiac arrest develop anxiety [64, 65] Assessing the striatal compartments in patients with hypoxic brain injury [66, 67] may reveal the neuroanatomical substrates for increased anxiety and depression in these individuals. Defining the contributions of each striatal compartment to anxiety and other neuropsychiatric disorders has the potential to reveal the distinct functions of striosome and matrix in human health and disease.

## Author Contributions

**Adrian T. Funk**: data acquisition and analysis, initial manuscript drafting, critical revision of the manuscript. **Jeff L. Waugh**: funding acquisition, supervision, software, data analysis and interpretation, critical manuscript revision.

## Funding

Jeff L. Waugh was supported by the CTSA Pilot Award; the Elterman Family Foundation; NINDS (Grant 1K23NS124978‐01A), the Brain and Behavior Research Foundation Young Investigator Award, and the Children’s Health CCRAC Early Career Award.

## Disclosure

All authors contributed to the article and approved the final version for submission. The content of this manuscript is solely the responsibility of the authors and does not necessarily represent the official views of these funding agencies.

## Conflicts of Interest

The authors declare no conflicts of interest.

## Data Availability

Publicly available datasets were analyzed in this study. NDA data can be accessed at nda.nih.gov. The code, bait, seed, and exclusion masks necessary to complete striatal parcellation can be accessed here github.com/jeff-waugh/Striatal-Connectivity-based-Parcellation.

## References

[1] Calhoon G. G. and Tye K. M. , Resolving the Neural Circuits of Anxiety, Nature Neuroscience. (2015) 18, no. 10, 1394–1404, 10.1038/nn.4101, 2-s2.0-84942474635. 26404714 PMC7575249

[2] Davis M. , Walker D. L. , Miles L. , and Grillon C. , Phasic vs. Sustained Fear in Rats and Humans: Role of the Extended Amygdala in Fear vs. Anxiety, Neuropsychopharmacology. (2010) 35, no. 1, 105–135, 10.1038/npp.2009.109, 2-s2.0-72049097678. 19693004 PMC2795099

[3] Lago T. , Davis A. , Grillon C. , and Ernst M. , Striatum on the Anxiety Map: Small Detours Into Adolescence, Brain Research. (2017) 1654, 177–184, 10.1016/j.brainres.2016.06.006, 2-s2.0-85002614346. 27276526 PMC5140771

[4] Han Y.-Y. , Jin K. , and Pan Q.-S. , et al.Microglial Activation in the Dorsal Striatum Participates in Anxiety-Like Behavior in Cyld Knockout Mice, Brain, Behavior, and Immunity. (2020) 89, 326–338, 10.1016/j.bbi.2020.07.011. 32688031

[5] Robinson O. J. , Overstreet C. , Charney D. R. , Vytal K. , and Grillon C. , Stress Increases Aversive Prediction Error Signal in the Ventral Striatum, Proceedings of the National Academy of Sciences. (2013) 110, no. 10, 4129–4133, 10.1073/pnas.1213923110, 2-s2.0-84874635488. PMC3593853 23401511

[6] Helfinstein S. M. , Benson B. , and Perez-Edgar K. , et al.Striatal Responses to Negative Monetary Outcomes Differ Between Temperamentally Inhibited and Non-Inhibited Adolescents, Neuropsychologia. (2011) 49, no. 3, 479–485, 10.1016/j.neuropsychologia.2010.12.015, 2-s2.0-79551500383. 21167189 PMC3065071

[7] Lee I. B. , Lee E. , and Han N.-E. , et al.Persistent Enhancement of Basolateral Amygdala-Dorsomedial Striatum Synapses Causes Compulsive-Like Behaviors in Mice, Nature Communications. (2024) 15, no. 1, 10.1038/s41467-023-44322-8, 219. PMC10774417 38191518

[8] Nikolaus S. , Antke C. , Beu M. , and Müller H.-W. , Cortical GABA, Striatal Dopamine and Midbrain Serotonin as the Key Players in Compulsive and Anxiety Disorders--Results From In Vivo Imaging Studies, Reviews in the Neurosciences. (2010) 21, no. 2, 119–139, 10.1515/REVNEURO.2010.21.2.119, 2-s2.0-77955060200. 20614802

[9] Graybiel A. M. and Hickey T. L. , Chemospecificity of Ontogenetic Units in the Striatum: Demonstration by Combining [3H]Thymidine Neuronography and Histochemical Staining, 79, *Proceedings of the National Academy of Sciences of the United States of America*, 1982, 198–202, 10.1073/pnas.79.1.198, 2-s2.0-0020077640. PMC345690 6172791

[10] Lévesque M. and Parent A. , The Striatofugal Fiber System in Primates: A Reevaluation of Its Organization Based on Single-Axon Tracing Studies, Proceedings of the National Academy of Sciences. (2005) 102, no. 33, 11888–11893, 10.1073/pnas.0502710102, 2-s2.0-23844446427. PMC1187973 16087877

[11] Kincaid A. E. and Wilson C. J. , Corticostriatal Innervation of the Patch and Matrix in the Rat Neostriatum, The Journal of Comparative Neurology. (1996) 374, no. 4, 578–592. 8910736 10.1002/(SICI)1096-9861(19961028)374:4<578::AID-CNE7>3.0.CO;2-Z

[12] Funk A. T. , Hassan A. A. O. , and Brüggemann N. , et al.In Humans, Striato-Pallido-Thalamic Projections are Largely Segregated by Their Origin in Either the Striosome-Like or Matrix-Like Compartments, Frontiers in Neuroscience. (2023) 17, 10.3389/fnins.2023.1178473, 1178473. 37954873 PMC10634229

[13] Sadiq A. , Funk A. T. , and Waugh J. L. , The Striatal Compartments, Striosome and Matrix, are Embedded in Largely Distinct Resting-State Functional Networks, Frontiers in Neural Circuits. (2025) 19, 10.3389/fncir.2025.1514937, 1514937. 40453419 PMC12122536

[14] Crittenden J. R. and Graybiel A. M. , Basal Ganglia Disorders Associated With Imbalances in the Striatal Striosome and Matrix Compartments, Frontiers in Neuroanatomy. (2011) 5, 10.3389/fnana.2011.00059, 2-s2.0-84859152259, 59. 21941467 PMC3171104

[15] Tippett L. J. , Waldvogel H. J. , and Thomas S. J. , et al.Striosomes and Mood Dysfunction in Huntington’s Disease, Brain. (2007) 130, no. 1, 206–221, 10.1093/brain/awl243, 2-s2.0-33845880405. 17040921

[16] Lawhorn C. , Smith D. M. , and Brown L. L. , Striosome-Matrix Pathology and Motor Deficits in the YAC128 Mouse Model of Huntington’s Disease, Neurobiology of Disease. (2008) 32, no. 3, 471–478, 10.1016/j.nbd.2008.08.006, 2-s2.0-56449119791. 18809498

[17] Mahmoudi S. , Samadi P. , and Gilbert F. , et al.Nur77 mRNA Levels and L-Dopa-Induced Dyskinesias in MPTP Monkeys Treated With Docosahexaenoic Acid, Neurobiology of Disease. (2009) 36, no. 1, 213–222, 10.1016/j.nbd.2009.07.017, 2-s2.0-69749086620. 19635563 PMC4807127

[18] Funk A. T. , Hassan A. A. O. , and Waugh J. L. , In Humans, Insulo-Striate Structural Connectivity is Largely Biased Toward Either Striosome-Like or Matrix-Like Striatal Compartments, Neuroscience Insights. (2024) 19, 10.1177/26331055241268079. PMC11402065 39280330

[19] Eblen F. and Graybiel A. M. , Highly Restricted Origin of Prefrontal Cortical Inputs to Striosomes in the Macaque Monkey, The Journal of Neuroscience. (1995) 15, no. 9, 5999–6013, 10.1523/JNEUROSCI.15-09-05999.1995. 7666184 PMC6577677

[20] Amemori K.-I. and Graybiel A. M. , Localized Microstimulation of Primate Pregenual Cingulate Cortex Induces Negative Decision-Making, Nature Neuroscience. (2012) 15, no. 5, 776–785, 10.1038/nn.3088, 2-s2.0-84860264805. 22484571 PMC3369110

[21] Karunakaran K. B. , Amemori S. , Balakrishnan N. , Ganapathiraju M. K. , and Amemori K.-I. , Generalized and Social Anxiety Disorder Interactomes Show Distinctive Overlaps With Striosome and Matrix Interactomes, Scientific Reports. (2021) 11, no. 1, 10.1038/s41598-021-97418-w, 18392. 34526518 PMC8443595

[22] Murray R. C. , Gilbert Y. E. , Logan A. S. , Hebbard J. C. , and Horner K. A. , Striatal Patch Compartment Lesions Alter Methamphetamine-Induced Behavior and Immediate Early Gene Expression in the Striatum, Substantia Nigra and Frontal Cortex, Brain Structure and Function. (2014) 219, no. 4, 1213–1229, 10.1007/s00429-013-0559-x, 2-s2.0-84904279773. 23625147 PMC3757105

[23] Friedman A. , Homma D. , and Gibb L. G. , et al.A Corticostriatal Path Targeting Striosomes Controls Decision-Making Under Conflict, Cell. (2015) 161, no. 6, 1320–1333, 10.1016/j.cell.2015.04.049, 2-s2.0-84930485706. 26027737 PMC4477966

[24] Canales J. J. and Graybiel A. M. , A Measure of Striatal Function Predicts Motor Stereotypy, Nature Neuroscience. (2000) 3, no. 4, 377–383, 10.1038/73949, 2-s2.0-0034069631. 10725928

[25] Aupperle R. L. , Sullivan S. , Melrose A. J. , Paulus M. P. , and Stein M. B. , A Reverse Translational Approach to Quantify Approach-Avoidance Conflict in Humans, Behavioural Brain Research. (2011) 225, no. 2, 455–463, 10.1016/j.bbr.2011.08.003, 2-s2.0-80052964729. 21843556 PMC3381365

[26] Hawes S. , Liang B. , and Oldham B. , et al.Patchy Striatonigral Neurons Modulate Locomotor Vigor in Response to Environmental Valence, ELife. (2025) 14, 10.7554/eLife.106403, RP106403. 41032042 PMC12488187

[27] Waugh J. L. , Hassan A. A. O. , and Kuster J. K. , et al.An MRI Method for Parcellating the Human Striatum Into Matrix and Striosome Compartments In Vivo, NeuroImage. (2022) 246, 10.1016/j.neuroimage.2021.118714. PMC9142299 34800665

[28] Holt D. J. , Graybiel A. M. , and Saper C. B. , Neurochemical Architecture of the Human Striatum, The Journal of Comparative Neurology. (1997) 384, no. 1, 1–25. 9214537 10.1002/(sici)1096-9861(19970721)384:1<1::aid-cne1>3.0.co;2-5

[29] Ragsdale C. W.Jr. and Graybiel A. M. , Compartmental Organization of the Thalamostriatal Connection in the Cat, Journal of Comparative Neurology. (1991) 311, no. 1, 134–167, 10.1002/cne.903110110, 2-s2.0-0025824162. 1719043

[30] Van Essen D. C. , Smith S. M. , Barch D. M. , Behrens T. E. J. , Yacoub E. , and Ugurbil K. , The WU-Minn Human Connectome Project: An overview, NeuroImage. (2013) 80, 62–79, 10.1016/j.neuroimage.2013.05.041, 2-s2.0-84880326067. 23684880 PMC3724347

[31] Busch E. L. , Slipski L. , and Feilong M. , et al.Hybrid Hyperalignment: A Single High-Dimensional Model of Shared Information Embedded in Cortical Patterns of Response and Functional Connectivity, NeuroImage. (2021) 233, 10.1016/j.neuroimage.2021.117975, 117975. 33762217 PMC8273921

[32] Hardi F. A. , Goetschius L. G. , and McLoyd V. , et al.Adolescent Functional Network Connectivity Prospectively Predicts Adult Anxiety Symptoms Related to Perceived COVID-19 Economic Adversity, Journal of Child Psychology and Psychiatry. (2023) 64, no. 6, 918–929, 10.1111/jcpp.13749. 36579796 PMC9880614

[33] Hubbard N. A. , Bauer C. C. C. , and Siless V. , et al.The Human Connectome Project of Adolescent Anxiety and Depression Dataset, Scientific Data. (2024) 11, no. 1, 10.1038/s41597-024-03629-x, 837. 39095370 PMC11297143

[34] Beck A. T. , Epstein N. , Brown G. , and Steer R. A. , An Inventory for Measuring Clinical Anxiety: Psychometric Properties, Journal of Consulting and Clinical Psychology. (1988) 56, no. 6, 893–897, 10.1037/0022-006X.56.6.893, 2-s2.0-0024245652. 3204199

[35] Flor H. , Fydrich T. , and Turk D. C. , Efficacy of Multidisciplinary Pain Treatment Centers: A Meta-Analytic Review, Pain. (1992) 49, no. 2, 221–230, 10.1016/0304-3959(92)90145-2, 2-s2.0-0026522788. 1535122

[36] Osman A. , Hoffman J. , Barrios F. X. , Kopper B. A. , Breitenstein J. L. , and Hahn S. K. , Factor Structure, Reliability, and Validity of the Beck Anxiety Inventory in Adolescent Psychiatric Inpatients, Journal of Clinical Psychology. (2002) 58, no. 4, 443–456, 10.1002/jclp.1154, 2-s2.0-0036196451. 11920696

[37] Ruiz M. A. , Zamorano E. , García-Campayo J. , Pardo A. , Freire O. , and Rejas J. , Validity of the GAD-7 Scale as an Outcome Measure of Disability in Patients With Generalized Anxiety Disorders in Primary Care, Journal of Affective Disorders. (2011) 128, no. 3, 277–286, 10.1016/j.jad.2010.07.010, 2-s2.0-78751635000. 20692043

[38] Seo J.-G. and Park S.-P. , Validation of the Generalized Anxiety Disorder-7 (GAD-7) and GAD-2 in Patients With Migraine, The Journal of Headache and Pain. (2015) 16, no. 1, 10.1186/s10194-015-0583-8, 2-s2.0-84947928729, 97. 26596588 PMC4656257

[39] Spitzer R. L. , Kroenke K. , Williams J. B. W. , and Löwe B. , A Brief Measure for Assessing Generalized Anxiety Disorder, Archives of Internal Medicine. (2006) 166, no. 10, 1092–1097, 10.1001/archinte.166.10.1092, 2-s2.0-33646815612. 16717171

[40] Kayikcioglu O. , Bilgin S. , Seymenoglu G. , and Deveci A. , State and Trait Anxiety Scores of Patients Receiving Intravitreal Injections, Biomedicine Hub. (2017) 2, no. 2, 1–5, 10.1159/000478993. PMC6945947 31988910

[41] Behrens T. E. J. , Berg H. J. , Jbabdi S. , Rushworth M. F. S. , and Woolrich M. W. , Probabilistic Diffusion Tractography With Multiple Fibre Orientations: What Can We Gain?, NeuroImage. (2007) 34, no. 1, 144–155, 10.1016/j.neuroimage.2006.09.018, 2-s2.0-33751096767. 17070705 PMC7116582

[42] Marecek S. , Krajca T. , and Krupicka R. , et al.Analysis of Striatal Connectivity Corresponding to Striosomes and Matrix in De Novo Parkinson’s Disease and Isolated REM Behavior Disorder, NPJ Parkinson’s Disease. (2024) 10, no. 1, 10.1038/s41531-024-00736-9, 124. PMC11199557 38918417

[43] Sadiq A. and Waugh J. L. , In Humans, fMRI Reveals That Striosome-Like and Matrix-Like Striatal Voxels are Engaged in Different Phases of Movement, Human Brain Mapping, 2026, 47, no. 3, 10.1002/hbm.70472. PMC12913692 41703732

[44] Flaherty A. W. and Graybiel A. M. , Corticostriatal Transformations in the Primate Somatosensory System. Projections From Physiologically Mapped Body-Part Representations, Journal of Neurophysiology. (1991) 66, no. 4, 1249–1263, 10.1152/jn.1991.66.4.1249, 2-s2.0-0026052743. 1722244

[45] Smith S. M. , Jenkinson M. , and Johansen-Berg H. , et al.Tract-Based Spatial Statistics: Voxelwise Analysis of Multi-Subject Diffusion Data, NeuroImage. (2006) 31, no. 4, 1487–1505, 10.1016/j.neuroimage.2006.02.024, 2-s2.0-33745188219. 16624579

[46] Benjamini Y. and Hochberg Y. , Controlling the False Discovery Rate: A Practical and Powerful Approach to Multiple Testing, Journal of the Royal Statistical Society Series B: Statistical Methodology. (1995) 57, no. 1, 289–300, 10.1111/j.2517-6161.1995.tb02031.x.

[47] Waugh J. L. , Hassan A. O. A. , Funk A. T. , and Maldjian J. A. , The Striatal Matrix Compartment is Expanded in Autism Spectrum Disorder, Journal of Neurodevelopmental Disorders. (2025) 17, no. 1, 10.1186/s11689-025-09596-7, 8. 39955485 PMC11829417

[48] Desban M. , Kemel M. L. , Glowinski J. , and Gauchy C. , Spatial Organization of Patch and Matrix Compartments in the Rat Striatum, Neuroscience. (1993) 57, no. 3, 661–671, 10.1016/0306-4522(93)90013-6, 2-s2.0-0027492966. 8309529

[49] Mikula S. , Parrish S. K. , Trimmer J. S. , and Jones E. G. , Complete 3D Visualization of Primate Striosomes by KChIP1 Immunostaining, Journal of Comparative Neurology. (2009) 514, no. 5, 507–517, 10.1002/cne.22051, 2-s2.0-66149117087. 19350670 PMC2737266

[50] Johnston J. G. , Gerfen C. R. , Haber S. N. , and van der Kooy D. , Mechanisms of Striatal Pattern Formation: Conservation of Mammalian Compartmentalization, Developmental Brain Research. (1990) 57, no. 1, 93–102, 10.1016/0165-3806(90)90189-6, 2-s2.0-0025241209. 1965303

[51] Goldman-Rakic P. S. , Cytoarchitectonic Heterogeneity of the Primate Neostriatum: Subdivision Into Island and Matrix Cellular Compartments, Journal of Comparative Neurology. (1982) 205, no. 4, 398–413, 10.1002/cne.902050408, 2-s2.0-0020026214. 7096628

[52] McGregor M. M. , McKinsey G. L. , Girasole A. E. , Bair-Marshall C. J. , Rubenstein J. L. R. , and Nelson A. B. , Functionally Distinct Connectivity of Developmentally Targeted Striosome Neurons, Cell Reports. (2019) 29, no. 6, 1419–1428.e5, 10.1016/j.celrep.2019.09.076. 31693884 PMC6866662

[53] Prager E. M. , Dorman D. B. , Hobel Z. B. , Malgady J. M. , Blackwell K. T. , and Plotkin J. L. , Dopamine Oppositely Modulates State Transitions in Striosome and Matrix Direct Pathway Striatal Spiny Neurons, Neuron. (2020) 108, no. 6, 1091–1102.e5, 10.1016/j.neuron.2020.09.028. 33080228 PMC7769890

[54] Crittenden J. R. , et al.Striosome-Dendron Bouquets Highlight a Unique Striatonigral Circuit Targeting Dopamine-Containing Neurons, Proceedings of the National Academy of Sciences of the United States of America. (2016) 113, no. 40, 11318–11323, 10.1073/pnas.1613337113, 2-s2.0-84989925957. 27647894 PMC5056098

[55] Friedman A. , Hueske E. , and Drammis S. M. , et al.Striosomes Mediate Value-Based Learning Vulnerable in Age and a Huntington’s Disease Model, Cell. (2020) 183, no. 4, 918–934.e49, 10.1016/j.cell.2020.09.060. 33113354 PMC7932131

[56] Friedman A. , Homma D. , and Bloem B. , et al.Chronic Stress Alters Striosome-Circuit Dynamics, Leading to Aberrant Decision-Making, Cell. (2017) 171, no. 5, 1191–1205.e28, 10.1016/j.cell.2017.10.017, 2-s2.0-85034116406. 29149606 PMC5734095

[57] Amemori S. , Amemori K. , and Yoshida T. , et al.Microstimulation of Primate Neocortex Targeting Striosomes Induces Negative Decision-Making, European Journal of Neuroscience. (2020) 51, no. 3, 731–741, 10.1111/ejn.14555, 2-s2.0-85073836297. 31429499 PMC7031011

[58] Weintraub D. , Newberg A. B. , and Cary M. S. , et al.Striatal Dopamine Transporter Imaging Correlates With Anxiety and Depression Symptoms in Parkinson’s Disease, Journal of Nuclear Medicine: Official Publication, Society of Nuclear Medicine. (2005) 46, no. 2, 227–232. 15695780

[59] Wang X. , Li J. , and Yuan Y. , et al.Altered Putamen Functional Connectivity is Associated With Anxiety Disorder in Parkinson’s Disease, Oncotarget. (2017) 8, no. 46, 81377–81386, 10.18632/oncotarget.18996, 2-s2.0-85030649468. 29113397 PMC5655292

[60] Tieu A. N. and Waugh J. L. , Depression-Associated Reductions in Putaminal Volume are Accompanied by a Shift From Matrix-Like to Striosome-Like Structural Connectivity, Frontiers in Psychiatry. (2025) 16, 10.3389/fpsyt.2025.1647240, 1647240. 40958791 PMC12434028

[61] Campbell J. S. W. and Pike G. B. , Potential and Limitations of Diffusion MRI Tractography for the Study of Language, Brain and Language. (2014) 131, 65–73, 10.1016/j.bandl.2013.06.007, 2-s2.0-84898889340. 23910928

[62] Fox M. D. , Liu H. , and Pascual-Leone A. , Identification of Reproducible Individualized Targets for Treatment of Depression With TMS Based on Intrinsic Connectivity, NeuroImage. (2013) 66, 151–160, 10.1016/j.neuroimage.2012.10.082, 2-s2.0-84870172400. 23142067 PMC3594474

[63] Vergallito A. , Gallucci A. , and Pisoni A. , et al.Effectiveness of Noninvasive Brain Stimulation in the Treatment of Anxiety Disorders: A Meta-Analysis of Sham or Behaviour-Controlled Studies, Journal of Psychiatry and Neuroscience. (2021) 46, no. 6, E592–E614, 10.1503/jpn.210050. 34753789 PMC8580831

[64] Chen X. , Li D. , and He L. , et al.The Prevalence of Anxiety and Depression in Cardiac Arrest Survivors: A Systematic Review and Meta-Analysis, General Hospital Psychiatry. (2023) 83, 8–19, 10.1016/j.genhosppsych.2023.03.013. 37028095

[65] Blennow Nordström E. , Vestberg S. , and Evald L. , et al.Neuropsychological Outcome After Cardiac Arrest: Results From a Sub-Study of the Targeted Hypothermia Versus Targeted Normothermia After Out-of-Hospital Cardiac Arrest (TTM2) Trial, Critical Care. (2023) 27, no. 1, 10.1186/s13054-023-04617-0, 328. 37633944 PMC10463667

[66] Sair H. I. , Hannawi Y. , and Li S. , et al.Early Functional Connectome Integrity and 1-Year Recovery in Comatose Survivors of Cardiac Arrest, Radiology. (2018) 287, no. 1, 247–255, 10.1148/radiol.2017162161, 2-s2.0-85044276899. 29043908

[67] Fink E. L. , Kochanek P. M. , and Beers S. R. , et al.Assessment of Brain Magnetic Resonance and Spectroscopy Imaging Findings and Outcomes After Pediatric Cardiac Arrest, JAMA Network Open. (2023) 6, no. 6, 10.1001/jamanetworkopen.2023.20713, e2320713. 37389874 PMC10314315

